# New York Odontological Society

**Published:** 1892-06

**Authors:** 

**Affiliations:** New York Odontological Society


### Reports of Society Meetings.

### NEW YORK ODONTOLOGICAL SOCIETY.

A REGULAR meeting of the New York Odontological Society was held on Tuesday evening, March 15, 1892, at the New York Academy of Medicine, No. 17 West Forty-third Street, with the president, Dr. Woodward in the chair.

The minutes of the previous meeting were read and approved.

INCIDENTS OF OFFICE PRACTICE.

*Dr. Bogue.*—I do not know what is new and what is old, but I will take the liberty to present a paint-box, furnished by Poulson, of Hamburg, containing eight colors, most of them oxides, and three bottles of oil, a box of platinum, brushes, etc. The object is to paint artificial teeth any color you need, and then bake it in. I had occasion to do something for a lady, who said, "Do not try to make me beautiful or the teeth beautiful." This is a specimen of what was done in the case, and I will pass it around. The colors I have not seen here. The upper part of this tooth has been painted and baked in afterwards. I imitated in a number of teeth the cavities and other defects that existed in the loose teeth that I had to extract.

Dr. Howe said he had not seen stearine used on oxyphosphate fillings. Here it is, and if any one wishes to help himself to a piece, he is welcome to it. It is to be flushed over the surface of the filling; it will polish somewhat more like enamel than the opaque phosphate filling.

The other day I needed a burnisher for a gold filling. I happened to find a glass bead of the proper shape; I placed it on an old engine burr, and had a beautiful instrument. I cemented with shellac, being sure the bead was hot enough to melt it.

Dr. John S. Marshall, of Chicago, Illinois, then read a paper, entitled, "Shock and Collapse resulting from Dental Operations."

(For Dr. Marshall's paper, see page 405.)

DISCUSSION.

*Dr. Francis.*—I have been waiting very patiently to hear some one speak upon this paper, which I consider a most excellent one, and contains many practical suggestions that come right home to us. I do not believe, and never have believed, in lengthy dental operations, and experience has taught me that very few people can sit more than an hour and a half or two hours in the dentist's chair without feeling much fatigue afterwards. They may not perceive it so much at the time as they are apt to do after they get home. I am in the habit of asking patients if they feel tired when operations are lengthy. Sometimes they say not, especially when they are anxious to have their work completed. When I consider that they have sat long enough, I send them home, and they often thank me for it afterwards.

I think we cannot be too careful in avoiding lengthy and painful operations for children. I rarely introduce gold fillings, and never very large ones, into the teeth of young children. I refer to the permanent teeth, of course; yet I have seen many temporary teeth filled with gold. Sixth-year molars are sometimes filled with gold soon after eruption, but in such cases they are liable to give out in a short time. They would do much better if filled with some plastic stopping. A lady in my office, one day, for whose child I was then operating, said to me that the child's eldest sister had previously quite a number of teeth filled by an excellent dentist. He did some very heroic work for her, and was anxious to have all the work done in a very limited time. After the operations were over, the child had St. Vitus's dance, and was for a long time in a very nervous condition.

*Dr. Jarvie.*—The paper is of such a character that one cannot but heartily recommend it. I am glad to see that he speaks not only of nervous strain upon the patient, but also of the nervous strain that the dentist is constantly subjected to. The experience that the essayist has had has been shared by most of us. I think that many dentists are often regardless of the tension upon the patient; the results are often exceedingly severe upon the middle-aged as well as the young.

In all operations upon children's teeth, the facility with which certain materials can be employed, even with only temporary benefit, ought to be considered; and often it is desirable to so treat the teeth as to tide them over a few months, and then repeat the operation, rather than subject a timid child to a long-continued nervous strain. A gentleman, of at least fifty years of age,—a man of iron nerve as well as muscle, a man who had an utter contempt for any kind of nervousness,—came to me one day. He had been a patient of mine for years, and would sit in the chair for hours, and submit to what would ordinarily be painful operations, with apparent indifference. His incisor teeth had worn away, and it seemed advisable to protect them, and also to lengthen the bite. Of course it involved sittings of some length, and he was quite desirous to spend every morning with me until the work would be completed. I told him he ought not to do it, that the strain would be too great. I remember his look of contempt when he said he could stand anything. I told him I thought I knew better, and said to him I would give him two mornings in succession, so as to get a good bearing, and then wait eight or ten days. He thought it was ridiculous, but I was immovable. The first sitting I capped two teeth, and then at the next I did two more. The operations were long, but they were not painful, yet he seemed to feel them more than he had in former times. He crawled out of my office without any sort of life or spirit, and he did not want to come near me for two weeks. That was a strong, well man. With children, and young ladies especially, the dentist should see that they do not have longer sittings than are necessary, and then have a sufficient time in which to recover from the strain before the next sitting.

Now, in regard to the dentist. I am a very earnest advocate of the desirability that the dentist should take a vacation from time to time, so as to keep himself in good condition. He ought not to allow himself to run down, constantly feeling fatigued and tired,—commencing in the morning with a feeling of almost terror at the day's work before him, and with a sense of intense relief when the day and its labors are over. A man in such a condition cannot do his best, and he is not doing justice either to himself or to his patients. Some, I know, think they are so busy that it is impossible for them to get away. They reason that if they were situated as some other people are they could do it. No one is too busy to get away. The busier a dentist is, the greater the necessity he will find for rest and fresh air occasionally, and that the world will get along without him for a time, possibly very much to his astonishment.

It may not be out of place to state my own practice, for few dentists work harder or enjoy better health. For years I have not been in my office on Saturday afternoons. Sometimes it is a long walk in the country; sometimes a drive; or it may be a saunter in New York City, looking at the shop-windows. In this way I not only get a relief from operating, but also the fresh air, and the change of thought and scene. At this season of the year, particularly, a dentist needs a vacation. He has been in his operating-room from the middle of September without any break in his regular work. With me there is a longing for fresh air that is irresistible, and, while it is as busy a season as any, yet next week I shall go away to be gone ten days, spending eight of them on the ocean, so that I can be in the air all the time. I get by this a degree of nervous force and energy that will allow me to more than make up for the lost time within six or eight weeks. I work with greater vim, and can do better work, on my return, than if I had stayed at home all the time. That is another way of recuperating. I think if we took longer vacations in the summer we could fill just as many teeth in the year, and have just as large an income. Get away from your ordinary surroundings. It has been my custom to take an ocean trip in the summer whenever practicable. I derive more benefit from a trip of that kind than from any other. It is a practice that is growing on professional men in this vicinity. You would be surprised to see the make-up of a passenger-list on one of the large steamers in the early part of July. I have met the same professional men—lawyers and physicians—year after year, the busiest men you can think of. They all tell the same story,—that they derive a benefit that they cannot get in any other way. We do not need rest exactly, but we want change,—change of air, change of thought, change of scene.

*Dr. Stockton.*—The one point of the essayist in regard to the filling of children's teeth should be emphasized. For years I have not filled the permanent teeth with gold, and it is the one thing that we should impress upon our patients, especially the parents. They say, " Are not these the permanent teeth ?" Yes, but gold is not the thing to fill them with. All of the teeth of my patients who have been treated in this manner are better preserved. We all know how much more exhausting it is to fill teeth for children than for adults.

I was also glad to hear the essayist speak of the proper recreation that we dentists should take. Do we really get the enjoy ment out of life that we should? We are doing harder work than almost any other class of men, and we get less from it. Let us say life is worth the living. We can do it if we will. No matter how much we accumulate here, we must leave it behind us.

*Dr. Howe.*—I think almost every dentist who has had some years of experience must have come to the conclusion that most of what Dr. Marshall has said is correct. But I suppose we are all apt to preach what we do not practise,—all apt to conclude that things are right that we do not quite follow. I must admit that I am frequently guilty of overtaxing the strength and nervous resistance of my patients, and I think it is a point that we should all bear in mind constantly. There is a tendency in practice for us to desire to get through with certain work, or to be over-persuaded by desire, on the part of the patient, to get through, and allow ourselves to be led to do those things which our deliberate judgment does not always approve. Let us keep in mind that we are not justified in injuring—even temporarily—our patients' health and strength in the effort to save their teeth.

*Dr. S. C. G. Watkins.*—I will only speak upon one point Dr. Marshall made,—that in regard to the dentist's rest after the noon meal. None of the other speakers have touched upon this, and I believe it to be a very important factor in the life of a busy operator. It is emphatically necessary for us to reserve our strength, and have regular time for certain work, and just as *regular time* for the much-required rest, in order that we may give our best efforts to our patients.

As a rule, when a dentist has been in practice for fifteen or twenty years, his nervous system becomes shattered from the constant strain which he is under from overwork. This state of things we must avoid. After working steadily for four or five hours, rest must be indulged in. A nap of half an hour is sufficient for a good reinforcement of nerve-power. This is my daily habit, and I consider it a grand investment. If we have regular working hours, and certain periods when we are absent from the office, our patients will soon understand that the engagements must be kept and will abide by them. I reserve every Tuesday afternoon for my own pleasure. I stop work at twelve o'clock and am through for the day. My regular patients do not come to my office on Tuesday any more than they would on Sunday. I think I work as hard as any man, but I could not do it without this rest. We can make our appointments so that we can spare time for a few days' outing. It is true, it is easier to preach than to practise, and we find difficulties in the way of taking these, but still, if we really want to we can, and we do our patients and ourselves a great injustice if we do not.

*Dr. S. E. Davenport.*—One of my neighbors has just whispered in my ear that this seems to him more like a Presbyterian prayer-meeting than a dental meeting. We cannot get up any fight, for there is no opposite side. I think Dr. Marshall is to be congratulated upon the arrangement of the expert testimony that he has given us to-night.

Reference was made, in the paper, to the greater liability of shock and collapse coming to a patient who was only partially anæsthetized than to one entirely under the influence of an anæsthetic. In the line of that thought, which I believe is a fact not disputed, comes to my mind the discussion at our last meeting upon the use of chloroform in dental practice. We were informed that if a patient should be given a few breaths of chloroform from a small vial, the effect, though slight, would suffice to prevent the great pain usually accompanying certain dental operation. There is, of course, no doubt but that chloroform will do, in almost every case, exactly what was said of it; but I was sorry that the lack of time prevented a proper expression of opinion on that important subject, for it seemed unfortunate that it should be allowed to go upon our proceedings almost without objection.

The gentleman who presented it did so in a questioning way, to be sure; and asked whether there were certain dangers connected with such use of the drug; but the fact that he was able to give as an authority for its use the names of two or three members of this society has given it a prominent and important position upon our proceedings, and unless we take pains to express ourselves to the contrary we really stand in the position of encouraging and recommending its use. The practice ought, in my opinion, to be strongly condemned; the drug is too powerful, and its effects too uncertain, for safe use in our operations, and we want nothing but remedies which are perfectly reliable.

*Dr. Bogue.*—We have all had this idea of shock floating before us in a misty way, but have never expressed it. Further than what the other gentlemen have stated I can say nothing. If I might turn to the question just broached,—that of partial anæsthesia,—I should like to inquire whether the evil effects of partial anæsthesia have been noticed, when it has been produced by chloroform administered so as not to strangle or suffocate the patient. One of my children said, the other day, that in passing by a house in my neighborhood, she had smelled chloroform in the street, and looking up to the window, had seen a surgical operation going on. It did not seem that that was the proper way to give any anæsthetic. The best way, I think, is as follows: I have stood as near as I am to my neighbor, and I could not detect the chloroform, the fact being that a little cylinder, about two and a half inches long, was attached to the mouth-piece, and on a sponge, not bigger than my thumb, was poured the chloroform, a few drops at a time. Whether the danger arises from the anæsthetic being given in that way, or given so that it almost suffocates the person, perhaps Dr. Marshall can tell us. I saw chloroform administered for partial anæsthesia from a little cylinder with very happy effects. I have not used it myself, and should hesitate to do so; but I imagine that that nice point of danger in the case is not yet very widely understood. Will Dr. Marshall tell us?

*Dr. Marshall.*—I think if Dr. Bogue would visit the hospitals, and ask to see the instrument-cases, he will find any quantity of inhalers that are never used now for administering chloroform. The vapor of chloroform is heavier than that of ether. It has a tendency to fall, and where you use an appliance which covers the face, the chances are that you will smother the patient, and almost all the surgeons that I have seen operating administer it on a napkin or on a handkerchief, so as to get all the atmospheric air possible into the lungs with the chloroform. The danger is in the amount which the patient gets at first. A large percentage of all the deaths have occurred within a minute or two after the commencement of the administration, showing that the patient has been overwhelmed, or that there was organic disease of the heart. The only safe way to administer chloroform is on a thin napkin or handkerchief.

*Dr. Bogue.*—The remark was made that a lady took a little chloroform from a bottle held in her hand, and death supervened. I do not know whether that is correct or not.

*Dr. Marshall.*—Quite likely; you will find that some people are affected more by chloroform than others, the result of idiosyncrasy. My wife was in the hands of my preceptor for the treatment of an exposed pulp. He used a bit of cotton saturated in chloroform to relieve the pain. Syncope immediately followed, and he worked over her for two hours before she was fit to go home. I would no more dare to give my wife chloroform than a dose of prussic acid, for I am convinced that death would follow in either case. This is an illustration of what I mean.

*Dr. Bogue.*—My last chloroform patient was one who took a Lubin's cologne bottle, and put some chloroform in it. She sat down in a chair, poured a few drops on her handkerchief, and took the same. While those idiosyncrasies exist, the question would arise in regard to other cases. There are a large number of these that we may expect would suffer no inconvenience.

*Dr. Marshall.*—I have for many years discarded the use of chloroform for dental operations. In my early practice I used chloroform frequently until I had to take a lady patient and hold her, head downward, to save her life. So I have used it sparingly since, except to little children for operations on the mouth in harelip or cleft palate. In these operations vomiting must be avoided, and chloroform gives the best results in this particular. In the use of ether, vomiting is likely to occur, even though you use morphia and atropia combined. Children take chloroform well, but the great danger from its use is the upright position. It is never safe in this position. The action of the chloroform is upon the brain. It produces anæmia of the brain, followed by syncope, and gradual paralysis of the pneumogastric nerve. When this stage is reached, the pumping force of the heart is materially lessened, and it cannot keep the brain supplied with blood, and the patient sinks. If the patient is laid on the table, gravitation carries the blood to the brain, and lessens the work of an already weakened condition of the heart's action. You may have read Dr. Meigs's criticism on the use of chloroform during labor. He says a great many patients insist upon sitting up as soon as consciousness is regained; the consequence is, they faint, heart clots are formed, and death supervenes.

*Dr. Watkins.*—I would like to ask Dr. Marshall a question. Is it not a fact that deaths occur more frequently where chloroform is administered in minor operations than in severe ones?

*Dr. Marshall.*—If you will say dental operations, in place of minor operations, I will answer yes. A large per cent. of the deaths under chloroform have been for minor operations, and a very large majority of them have been dental operations. For the mere lancing of an abscess under chloroform, if the patient is lying down, there is comparatively little danger. It is the upright position in these cases that does the harm. Profound anæsthesia is less dangerous than partial for any operation.

*Dr. Jarvie.*—I would like to ask Dr. Marshall if he would consider himself justified in using chloroform in dental operations?

*Dr. Marshall.*—I do not administer chloroform unless my patient is lying flat upon the table or bed. And rarely use it except for operations upon little children and those cases in which there is serious disease of the kidneys.

*Dr. Dwinelle.*—When chloroform was first introduced, we gave it for all kinds of operations, for minor as well as severe ones, and considered ourselves safe in doing it; but, latterly, chloroform seems to have exceptions to its rules and almost to have become an idiosyncrasy itself. I think I referred to a certain case I had the other day. It became very important that I should extract a tooth for a lady, the wife of a physician, and she insisted upon taking chloroform. I felt justified, under the circumstances, in coinciding with her, although her husband was opposed to it. We gave it to her. The last time her husband had seen chloroform administered was to a young lady who had died from its effects. The operation on his wife was successful. Chloroform has either differed in its character or in its effects. I notice abroad, out of compliment to Dr. Simpson, of Edinburgh, the discoverer, chloroform is used very generally, while ether is not so much as here. One is regarded as a European affair, and the other as an American. I never hesitate about using chloroform when it is indicated, but always, of course, with precaution and having proper remedies at hand.

I want to say a word in regard to the paper, which I commend most heartily. None of us could question its teachings; it is self-evident in all of its propositions. We need more recreation; we need to avoid the shocks upon patients and upon ourselves. We need more rest; and that reminds me of what Dr. Marshall told me this very evening,—that Dr. Allport takes a half-hour's rest at one o'clock every day, no matter what the circumstances that surround him. He has the happy faculty of dropping to sleep for half an hour, and waking up refreshed. With reference to what we may derive from our patients, and the disadvantages of contact that we are subjected to, it occurs to me how many of our friends in the profession have died from cancer of the stomach.

*Dr. Marshall.*—There is one thing I forgot to say about chloroform. In my hospital work, a great many cases are there for other troubles, and when they require my services I have the patient laid on the operating-table, and extract the teeth. I have no trouble when they lie that way, but there is an exception to the use of ether in certain cases, and that is where we have kidney troubles, as Bright's disease or diabetes. If it were a small operation, I should use neither ether nor chloroform, but nitrous oxide; but if a long operation upon such a subject, I should prefer chloroform, and not think of ether.

On motion of Dr. Francis, the thanks of the Society were tendered to Dr. Marshall for his kindness in coming so far to present this very able paper.

*Dr. R. C. Brewster.*—I desired to get some information in regard to the sulphate of morphia, which, I think, the doctor spoke of having used to help patients endure long operations. If I heard correctly, he said he was in the habit of giving a quarter of a grain. I would like to ask how he uses it, hypodermically or by the stomach.

*Dr. Marshall.*—By the stomach, with whiskey.

*Dr. Brewster.*—At the last meeting a gentleman, who was a member of the Society, made the statement that he was in the habit, whenever he saw a patient looking pale, of giving a hypodermic injection of that amount of sulphate of morphia without the slightest hesitation, and making no distinction. That may be all right; and I think the practice no doubt is good in the hands of men who are able to make proper discriminations; but perhaps it would be well if some one would raise the voice of admonition against its use, as it is such a deadly drug. Will Dr. Marshall tell us as to when not to use it?

*Dr. Marshall.*—I should not give that amount to a child. I mentioned one case in my paper of a lady who was a frail, delicate woman, about twenty-seven years old, to whom I gave it regularly. To some people it would not be necessary to give more than an eighth of a grain, but no harm could come from even a quarter, administered in that way. One-half grain is the maximum dose, and no one should take more. A quarter of a grain will make most people drowsy, but that is the effect we want.

Adjourned.

JOHN I. HART, D.D.S.,
*Editor New York Odontological Society.*

